# STRENGTH IN AGE: HARNESSING THE BENEFITS OF NIA’S 45 YEARS

**DOI:** 10.1093/geroni/igz038.1638

**Published:** 2019-11-08

**Authors:** Marie A Bernard, Richard Hodes, Robin A Barr

**Affiliations:** National Institute on Aging, National Institutes of Health, Bethesda, Maryland, United States

## Abstract

The National Institute on Aging at the National Institutes of Health was established in 1974 with a mission to support and conduct research on aging processes, age-related diseases, and special problems and needs of the aged. Shortly thereafter, the Institute was designated by Congress as the lead research agency in Alzheimer’s disease. During this symposium, NIA’s scientific leadership will reflect on major accomplishments to improve the health of older individuals over the past 45 years and look to the future of the NIA and aging research. This symposium will describe progress led by NIA’s extramural divisions, the intramural program, and the Office of Special Populations. A brief panel discussion will ensue, followed by an informal reception and opportunities for networking in celebration of NIA’s 45 years

